# Diabetes-Driven Atherosclerosis: Updated Mechanistic Insights and Novel Therapeutic Strategies

**DOI:** 10.3390/ijms26052196

**Published:** 2025-02-28

**Authors:** Paschalis Karakasis, Panagiotis Theofilis, Dimitrios Patoulias, Panayotis K. Vlachakis, Antonios P. Antoniadis, Nikolaos Fragakis

**Affiliations:** 1 Second Department of Cardiology, Medical School, Hippokration General Hospital, Aristotle University of Thessaloniki, Konstantinoupoleos 49, 54124 Thessaloniki, Greece; aantoniadis@gmail.com (A.P.A.); fragakis.nikos@googlemail.com (N.F.); 2 First Cardiology Department, School of Medicine, Hippokration General Hospital, National and Kapodistrian University of Athens, 15772 Athens, Greece; panos.theofilis@hotmail.com (P.T.); vlachakispanag@gmail.com (P.K.V.); 3 Second Propedeutic Department of Internal Medicine, Faculty of Medicine, School of Health Sciences Aristotle, University of Thessaloniki, 54124 Thessaloniki, Greece; dipatoulias@gmail.com

**Keywords:** atherosclerosis, diabetes, inflammation, oxidative stress, hyperglycemia, epigenetic regulation, precision medicine

## Abstract

The global rise in diabetes prevalence has significantly contributed to the increasing burden of atherosclerotic cardiovascular disease (ASCVD), a leading cause of morbidity and mortality in this population. Diabetes accelerates atherosclerosis through mechanisms such as hyperglycemia, oxidative stress, chronic inflammation, and epigenetic dysregulation, leading to unstable plaques and an elevated risk of cardiovascular events. Despite advancements in controlling traditional risk factors like dyslipidemia and hypertension, a considerable residual cardiovascular risk persists, highlighting the need for innovative therapeutic approaches. Emerging treatments, including sodium–glucose cotransporter 2 (SGLT2) inhibitors, glucagon-like peptide-1 (GLP-1) receptor agonists, epigenetic modulators, and RNA-based therapies, are showing promise in addressing the unique challenges of diabetes-associated ASCVD. Precision medicine strategies, such as nanoparticle-based drug delivery and cell-specific therapies, offer further potential for mitigating cardiovascular complications. Advances in multiomics and systems biology continue to deepen our understanding of the molecular mechanisms driving diabetes-associated atherosclerosis. This review synthesizes recent advances in understanding the pathophysiology and treatment of diabetes-related atherosclerosis, offering a roadmap for future research and precision medicine approaches to mitigate cardiovascular risk in this growing population.

## 1. Introduction

The global prevalence of diabetes is increasing at an alarming rate, posing a significant public health challenge and placing millions at risk of its devastating complications [1]. Among these, atherosclerotic cardiovascular disease (ASCVD) remains the leading cause of morbidity and mortality in individuals with diabetes [2]. The intersection of diabetes and atherosclerosis is particularly concerning, as diabetes not only accelerates atherosclerotic plaque formation and destabilization but also often coexists with other risk factors [3,4], significantly increasing the likelihood of adverse cardiovascular events such as myocardial infarction, stroke, and peripheral artery disease.

The pathogenesis of diabetes-associated atherosclerosis is multifactorial, driven by hyperglycemia, chronic inflammation, oxidative stress, and profound metabolic disturbances [5]. Despite advances in managing traditional cardiovascular risk factors such as dyslipidemia, hypertension, and hyperglycemia, a substantial residual risk persists in this population, underscoring the need for novel therapeutic approaches. Recent research highlights additional layers of complexity, including the roles of epigenetic modifications, immune system dysregulation, and cell-specific dysfunction in the progression of atherosclerosis in diabetes.

This review explores the unique mechanisms underlying atherosclerosis in diabetes and highlights emerging therapeutic strategies aimed at mitigating these risks. Innovations such as epigenetic modulators, RNA-based therapies, nanomedicine, and advanced glucose-lowering agents offer promising avenues for addressing the complex interplay of metabolic, inflammatory, and vascular factors contributing to ASCVD in diabetes. By synthesizing recent advances, this study aims to provide a framework for future research and guide the development of targeted therapies to improve cardiovascular outcomes in this high-risk and growing patient population.

## 2. Mechanisms Mediating Atherosclerotic Risk

**Figure 1:**
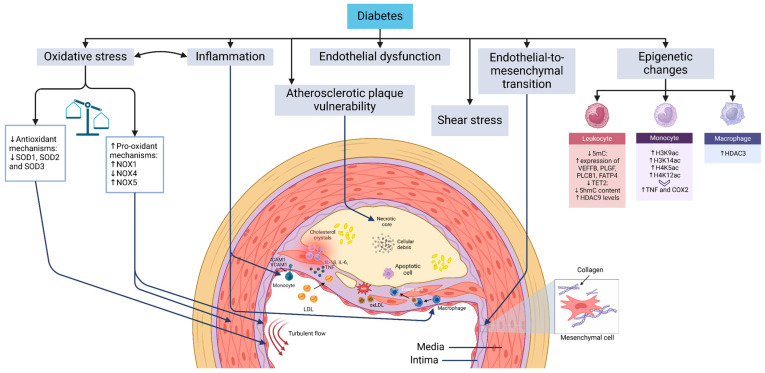
Mechanisms driving atherosclerosis development and progression in diabetes. Diabetes mellitus is closely linked to pathogenic mechanisms that are integral to the development of atherosclerosis. Turbulent blood flow sensitizes endothelial cells at specific vascular sites, enabling them to exhibit distinct responses to systemic risk factors such as hypercholesterolemia and hyperglycemia. While the progression of atherosclerotic plaque formation follows a similar trajectory in individuals with and without diabetes, the process of atherogenesis is significantly accelerated in the context of diabetes. Key mechanisms contributing to plaque formation include endothelial dysfunction, endothelial-to-mesenchymal transition, the transmigration of monocytes across the endothelium followed by their differentiation into macrophages, the formation of foam cells, and the proliferation and migration of vascular smooth muscle cells toward the fibrous cap of the plaque. Abbreviations: COX2 (cyclooxygenase-2), H3K14ac (histone 3 lysine 14 acetylation), H3K9ac (histone 3 lysine 9 acetylation), H4K12ac (histone 4 lysine 12 acetylation), H4K5ac (histone 4 lysine 5 acetylation), HDAC (histone deacetylase), ICAM1 (intercellular adhesion molecule 1), NOX (NADPH oxidase), oxLDL (oxidized LDL), ROS (reactive oxygen species), ShmC (5-hydroxymethylcytosine), SmC (5-methylcytosine), SOD (superoxide dismutase), TET2 (methylcytosine dioxygenase TET2), TNF (tumor necrosis factor), and VCAM1 (vascular cell adhesion protein 1).

ASCVD in diabetes presents with key manifestations such as myocardial infarction, peripheral artery disease, and stroke [6,7]. Understanding the underlying mechanisms and the impact of glucose-lowering therapies on cardiovascular outcomes is critical for effective management [8]. Diabetes accelerates atherosclerosis through pathways shared with non-diabetic individuals, including endothelial dysfunction, shear stress-induced vascular changes, endothelial-to-mesenchymal transition (EndMT), monocyte infiltration, macrophage and foam cell formation, and vascular smooth muscle cell (VSMC) proliferation (Figure 1). Advances in research and imaging have identified novel mechanisms that could offer new therapeutic opportunities.

### 2.1. Oxidative Stress

Oxidative stress, characterized by an imbalance between excessive reactive oxygen species (ROS) production and insufficient antioxidant defenses, plays a critical role in the pathogenesis of diabetes-related atherosclerosis [9,10,11]. Major sources of ROS within the vascular wall include the mitochondrial electron transport chain, NADPH oxidases (NOX), xanthine oxidase, and endothelial nitric oxide synthase (eNOS) [11]. While ROS serve beneficial roles, such as facilitating the respiratory burst in neutrophils for bacterial clearance, their overproduction leads to detrimental vascular effects [9].

In endothelial cells, hyperglycemia induces elevated mitochondrial ROS production [12,13]. This occurs due to increased glucose metabolism within mitochondria, which drives hexosamine and polyol pathway flux, enhances the formation of advanced glycation end product (AGE) precursors (e.g., methylglyoxal), and activates protein kinase C [14]. Experimental studies demonstrate that overexpression of mitochondrial uncoupling protein 1 or superoxide dismutase 2 (an antioxidant enzyme) effectively prevents mitochondrial superoxide production [15], highlighting a potential link between hyperglycemia and microvascular complications [16]. While epidemiological evidence suggests a clear association between hyperglycemia and macrovascular disease, the mechanistic pathways are less well defined compared to microvascular disease [17]. However, components of metabolic syndrome, including insulin resistance and increased fatty acid oxidation, further exacerbate ROS production from mitochondrial and other sources, contributing to the progression of diabetes-associated atherosclerosis.

The diabetic milieu, characterized by hyperglycemia, toxic glucose-derived metabolites, elevated systemic and local levels of AGEs, and activation of pathways such as the AGE receptor, the renin–angiotensin–aldosterone system, and growth factors, is strongly associated with heightened ROS production via NOX enzymes [9,10,11]. Among these, NOX1 plays a pivotal role in diabetes-driven atherosclerosis. In Apoe+ mice with STZ-induced diabetes, genetic deletion of Nox1 or pharmacological inhibition using GKT137831 (setanaxib, a dual NOX1/NOX4 inhibitor) significantly reduced atherosclerotic burden [18], as evidenced by decreased plaque area and improved plaque morphology [19]. Conversely, global deletion of Nox4 was linked to enhanced plaque formation and increased plaque complexity [20], characterized by heightened collagen deposition and altered extracellular matrix composition. Subsequent research revealed that NOX4 exerts vasculoprotective effects [21], potentially through its production of H_2_O_2_, which can be vasodilatory or pro-atherogenic depending on its localization and concentration. Distinct roles of NOX1 and NOX4 in modulating immune cell composition and VSMC morphology within atherosclerotic plaques in diabetes have been demonstrated in later studies [22,23].

Studies have demonstrated that the antioxidant defense mechanisms are diminished in diabetes-associated atherosclerosis, and restoring these defenses can mitigate plaque formation in murine models [24]. However, pharmacological targeting of certain antioxidant pathways, such as nuclear factor erythroid 2-related factor 2 (Nrf2), has proven challenging due to limited efficacy or significant off-target effects, particularly at higher doses [25]. Consequently, a more viable strategy appears to be the direct inhibition of NOX enzymes, which represent a primary source of ROS.

Over the past years, research has increasingly focused on NOX5, an isoform expressed in humans but absent in rodents. In a mouse model of diabetic kidney disease, transgenic expression of human NOX5 in endothelial cells (via the vascular endothelial cadherin promoter) or VSMCs (via the transgelin promoter) accelerated renal injury compared with controls [26,27]. However, the impact of NOX5 overexpression in atherosclerosis models remains less definitive [28,29]. Studies using a different promoter (Tie2) to drive NOX5 expression in endothelial cells found that NOX5 was linked to increased aneurysm formation in diabetic mice [29] and hypertension in non-diabetic mice [28], but it did not result in a significant increase in atherosclerotic plaque area [29]. In human coronary artery plaques, NOX5 shows elevated expression in endothelial cells during early atherosclerosis and in both endothelial cells and VSMCs in advanced coronary artery disease [30].

### 2.2. Blood Flow and Shear Stress

Atherosclerosis tends to develop at specific regions of blood vessels influenced by hemodynamic forces, particularly shear stress, which represents the frictional force of blood flow on the endothelial layer [31]. Plaques form preferentially at arterial branches, bends, and bifurcations—areas subjected to turbulent blood flow with oscillatory low shear stress [32]. In contrast, straight arterial segments exposed to stable laminar flow with high shear stress are typically resistant to atherosclerosis development [31]. Endothelial cells are particularly susceptible to damage in diabetes due to their continuous exposure to both hemodynamic forces and metabolic stressors [33]. While transmural and circumferential stress caused by blood pressure predominantly impacts VSMCs in the vascular media, shear stress resulting from blood flow primarily affects the endothelium [33]. This exposure leads to profound structural, functional, transcriptional, and epigenomic alterations in endothelial cells, further exacerbating their vulnerability [33].

Mechanosensory complexes on the endothelial cell membrane act as sensors for varying blood flow patterns, converting mechanical stimuli into intracellular signals through mechanotransduction pathways [34]. Activation of these pathways modulates the activity and expression of transcription factors and epigenetic regulators, such as epigenetic enzymes and non-coding RNAs, eliciting cellular responses that influence endothelial function [31,35]. While hemodynamic forces associated with blood flow do not initiate atherosclerotic plaque formation, they prime endothelial cells at specific vascular sites to respond uniquely to systemic risk factors, including hypercholesterolemia and hyperglycemia [36]. At regions subjected to turbulent, low shear stress and oscillatory blood flow, endothelial cells undergo reprogramming, resulting in inflammation, oxidative stress, dysfunction, and EndMT [37]. In diabetes, these pro-atherogenic pathways are significantly exacerbated by the hyperglycemic and pro-inflammatory environment, accelerating disease progression [5,38,39]. The cardioprotective effects of sodium–glucose cotransporter 2 (SGLT2) inhibitors, such as empagliflozin, extend beyond their glucose-lowering properties [40]. Evidence from a non-randomized, open-label, prospective cohort study suggests that empagliflozin exerts direct vascular effects by increasing blood viscosity and wall shear stress when compared to incretin-based therapies in patients with type 2 diabetes [40]. Furthermore, treatment with empagliflozin was associated with a significant and early reduction in intima–media thickness, highlighting its potential role in mitigating vascular pathology independently of glycemic control [41].

Blood flow-associated molecular mechanisms, including flow-sensitive transcription factors and epigenetic pathways, have emerged as promising therapeutic targets for atherosclerosis [36]. Advances in cell-specific multiomics approaches and the development of in vitro shear stress models—such as microfluidic devices, parallel-plate chambers, and cone-and-plate viscometers—have facilitated the identification of novel endothelial cell-specific therapeutic targets [36]. These targets are related to key processes such as oxidative stress, EndMT, and inflammation [35,36]. Extensive research has generated a comprehensive list of flow-sensitive genes, as summarized in various reviews. Among the most therapeutically relevant genes are those associated with anti-atherogenic properties (e.g., KLF2, KLF4, NOS3, and NOX4), antioxidant defense (e.g., SOD2 and SOD3), and pro-atherogenic effects (e.g., genes within the activator protein 1 (AP-1) complex, CCL2, ICAM1, VCAM1, NFKB, NOX1, NOX2, MMPs, MAPK1, MAPK3, and HIF1A) [42]. These findings, validated through in vivo animal models and human studies, highlight critical molecular targets with potential for translational therapies in atherosclerosis. Of note, in a type 2 diabetes mouse model, physical exercise-induced KLF2 expression was shown to activate endothelial nitric oxide synthase (eNOS/NOS3), resulting in improved vasodilation [43]. Additionally, a study employing RNA sequencing and NanoString technology to analyze the gene expression profiles of atherosclerotic plaques in pigs and humans, both with and without diabetes, identified KLF4 as a key gene with a distinct expression pattern associated with the progression of atherosclerosis in the context of diabetes [44].

Several of the aforementioned pro-atherogenic genes, such as ICAM1, VCAM1, and NOX1, have been shown to be upregulated in diabetes-associated atherosclerosis in both human studies and animal models [19,45]. Research has demonstrated that NADPH oxidase 1 (NOX1) acts as a pro-atherogenic and pro-oxidant factor, while NOX4 exhibits atheroprotective properties in STZ-induced diabetic Apoe+ mice, a widely used model for studying diabetes-associated atherosclerosis [19,20]. Furthermore, studies in the same model revealed that atheroprone regions of the aorta subjected to turbulent blood flow exhibited elevated activator protein 1 (AP-1) activity compared with regions exposed to laminar flow [46]. In an in vitro microfluidics-based model simulating pro-atherogenic low shear stress in human aortic endothelial cells (HAECs), high glucose concentrations significantly increased AP-1 activity and expression compared with conditions of high shear stress in the absence of hyperglycemia [46]. Similarly, elevated AP-1 levels were observed in carotid endarterectomy specimens from patients with diabetes compared with those from non-diabetic individuals [46]. Flow-sensitive epigenetic targets implicated in these processes include microRNAs (e.g., miR-10a, miR-19a, miR-23b, miR-205, and miR-21), long non-coding RNAs (e.g., MALAT1, STEEL, LASSIE, and LISPRI), DNA methyltransferases (DNMT1 and DNMT3), histone deacetylases (HDAC1-HDAC7 and SIRT1), and histone methyltransferases (e.g., EZH2) [34,47,48,49,50]. Therapeutic strategies could involve either promoting stable flow-induced atheroprotective pathways or suppressing turbulent flow-mediated pro-atherogenic mechanisms [48,49,50]. These goals may be achieved through small-molecule inhibitors, recombinant proteins, or, preferably, site-specific drug delivery systems to maximize therapeutic precision and efficacy [48,49,50].

### 2.3. Atherosclerotic Plaque Vulnerability

Diabetes is associated not only with an increased atherosclerotic plaque burden but also with the development of unstable plaques, which are highly susceptible to rupture [51]. These plaques are characterized by features such as enlarged necrotic cores, intraplaque hemorrhage, and thin fibrous caps, contributing to their instability [51]. Clinically, this heightened plaque vulnerability in diabetes correlates with an increased incidence of adverse cardiovascular events, including myocardial infarction and stroke, in individuals with both diabetes and atherosclerotic CVD compared to those without diabetes [52].

A mouse model replicating unstable atherosclerotic plaques has been established using the tandem stenosis method, where two ligations are applied to the left carotid artery, inducing plaque characteristics that closely resemble those observed in humans [51]. Under hyperglycemic conditions induced by STZ, these mice exhibited greater plaque instability, marked by larger necrotic cores, elevated macrophage infiltration, intraplaque hemorrhage, a reduced fibrous cap-to-plaque size ratio, increased collagen deposition, and decreased NOX4 expression [51]. Importantly, early intervention with the SGLT2 inhibitor dapagliflozin, initiated five weeks post-diabetes induction and three days post-tandem stenosis, mitigated these pathological changes, providing proof-of-concept evidence that SGLT2 inhibitors exert vasculoprotective and anti-atherosclerotic effects partially independent of their glucose-lowering properties [51]. These benefits have since been validated in clinical studies, underscoring the therapeutic potential of SGLT2 inhibitors in managing diabetes-associated CVD [53].

Additional mechanisms contributing to the formation of vulnerable atherosclerotic plaques in diabetes have been identified. In a mouse model of type 1 diabetes, the use of antisense oligonucleotides targeting Apoc3 effectively reduced necrotic core size [54]. Inflammasome activation also appears to play a significant role. Studies in atherosclerosis-prone mice demonstrated that deficiency of NLRP3, AIM2 (absent in melanoma 2), or gasdermin D in hematopoietic cells was associated with a reduction in overall atherosclerotic lesion size. However, these deficiencies did not prevent necrotic core formation, suggesting that the reduction in lesion size is independent of macrophage pyroptosis [55]. Moreover, pharmacological inhibition of NLRP3 using MCC950, a specific NLRP3 inhibitor, significantly decreased both atherosclerotic plaque size and necrotic core formation in Apoe+ mice with STZ-induced diabetes, highlighting NLRP3 as a potential therapeutic target in diabetes-associated atherosclerosis [56].

### 2.4. Endothelial-to-Mesenchymal Transition

EndMT is a complex, dynamic process wherein endothelial cells transition into mesenchymal-like cells, such as myofibroblasts and VSMCs, involving extensive molecular and phenotypic changes [57,58]. During this transition, endothelial cells lose their distinct characteristics, such as apical-to-basal polarity and cell–cell adhesion, and acquire mesenchymal traits, including migratory capability and spindle-shaped morphology [57,58]. Molecularly, this process is marked by the downregulation of endothelial markers (e.g., vascular endothelial cadherin and von Willebrand factor) and upregulation of mesenchymal markers (e.g., TAGLN, calponin, and αSMA) [59].

EndMT plays a vital role during embryogenesis in processes like cardiogenesis and vasculogenesis [38]. However, aberrant induction by environmental factors such as hyperglycemia, turbulent blood flow, oxidized LDL (oxLDL), hypoxia, oxidative stress, and chronic inflammation contributes significantly to diabetes-accelerated atherosclerosis. Studies using lineage-tracking and Cre-lox systems in mouse models have identified EndMT-derived fibroblast-like cells within atherosclerotic plaques [60,61,62], and single-cell RNA sequencing has confirmed the presence of endothelial subpopulations expressing mesenchymal markers in both human and mouse plaques [63,64,65,66]. These findings suggest that EndMT is a continuum, with cells often existing in a “partial” or intermediate EndMT state, co-expressing both endothelial and mesenchymal markers [67,68,69,70].

Therapeutic strategies targeting EndMT in atherosclerosis have shown promise. For instance, inhibiting the TGFβ signaling pathway, a critical driver of EndMT, using endothelial-specific siRNA delivered via lipid nanoparticles suppressed EndMT and reversed atherosclerosis in preclinical models [71,72,73]. Epigenetic regulation, such as DNA methylation and histone modifications, has also been implicated in EndMT [74]. In mice, endothelial-specific deletion of Hdac9 reduced EndMT, decreased lesion size, and enhanced plaque stability [75].

Although EndMT is commonly observed in diabetes-related complications, its modulation in diabetes-associated atherosclerosis remains underexplored [76,77]. Studies in type 2 diabetes models, including db/db mice and patients with diabetes, have demonstrated EndMT in the aorta, evidenced by co-expression of CD31 and αSMA, as well as increased expression of transcription factors such as SLUG, TWIST, and SNAIL [76,78]. Moreover, Kruppel-like factor 7 has been identified as a target of miR-132-3p and is implicated in hyperglycemia-induced EndMT [76]. Experimental overexpression of miR-132-3p in human endothelial cells reduced KLF7 levels and ameliorated EndMT [76]. Additionally, transcription factors such as SMAD2, SMAD3, RHO-associated kinase 1, and serum response factor have been shown to regulate mesenchymal marker expression during EndMT [79,80].

In vitro studies with human endothelial cells under hyperglycemic conditions have revealed that multiple signaling pathways and transcription factors, including SMAD, angiotensin II, endothelin 1, poly(ADP-ribose) polymerase 1, and extracellular signal-regulated kinase, drive EndMT [81,82]. These findings highlight the potential of targeting EndMT-related pathways as a therapeutic strategy for diabetes-associated atherosclerosis, but further research is needed to explore clinical applications.

### 2.5. Epigenetic Alterations

Gene transcription is tightly regulated through multiple pathways, including epigenetic mechanisms that respond dynamically to environmental stimuli. Epigenetic modifications at regulatory gene regions can alter gene expression, and dysregulation of these processes is implicated in tissue damage and the progression of diseases such as atherosclerosis [83].

The epigenetic landscape plays a central role in establishing and maintaining chromatin structure, which is crucial for nuclear functions like DNA replication, repair, and gene expression [84]. Chromatin’s structural state—condensed or open—determines gene accessibility, with condensed chromatin suppressing transcription and open chromatin facilitating it [84]. Key enzymes involved in epigenetic regulation include those mediating DNA methylation, histone modifications, and non-coding RNA pathways. These epigenetic processes are increasingly recognized as significant contributors to the pathophysiology of cardiovascular diseases (CVD), including those associated with diabetes, highlighting their potential as therapeutic targets [85].

### 2.6. DNA Methylation

DNA methylation, an enzymatic process involving the addition of a methyl group to cytosine at CpG sites, is a critical mechanism of epigenetic gene regulation [86]. At regulatory regions, DNA methylation can silence gene expression either directly by inhibiting transcription factor binding or indirectly by altering chromatin remodeling protein affinity [87,88,89,90]. Aberrant DNA methylation has been implicated in key processes driving atherosclerosis, including inflammation, endothelial dysfunction, foam cell formation, and VSMC proliferation [91,92]. However, its role in diabetes-associated atherosclerosis remains poorly understood [93,94].

A study using methylation profiling identified hypomethylation of genes such as VEGFB, PLGF, PLCB1, and FATP4 as being associated with CVD in individuals with diabetes [95]. These findings were validated using larger cohorts and advanced techniques, such as methylation-specific PCR [95]. Despite promising preclinical studies showing atheroprotective effects of DNMT inhibitors (e.g., 5-azacytidine and 5-aza-2-deoxycytidine) [96], which are approved for myelodysplasia treatment, clinical trials for atherosclerotic CVD remain absent due to the systemic off-target effects of DNMT inhibition [97,98]. Targeted delivery using nanocarriers to limit DNMT inhibition to specific cell types is a potential solution to this limitation.

DNA demethylation, mediated by enzymes such as TET2, plays an equally critical role in atherosclerosis. TET2 oxidizes 5-methylcytosine to 5-hydroxymethylcytosine (5 hmC), a process implicated in inflammatory pathways, endothelial dysfunction, and diabetic complications like retinopathy [99,100,101,102]. TET2 also plays a central role in clonal hematopoiesis of indeterminate potential (CHIP), a process where somatic mutations in hematopoietic cells lead to clonal expansion, independently increasing the risk of atherosclerotic CVD [103]. In diabetes, reduced 5 hmC levels and diminished TET2 activity in circulating leukocytes have been observed, contributing to increased myelopoiesis, atherogenic monocyte production, and accelerated atherosclerosis [104]. Preclinical studies have shown promise in targeting CHIP and TET2-related pathways [105,106]. For example, vitamin C has been shown to restore TET2 activity and hematopoiesis in Tet2+ mice [106], while targeting sirtuin 1 has been demonstrated to influence TET2 function in hematopoietic stem cells [105].

### 2.7. Histone Modifications

Histones, the protein components of chromatin, undergo various covalent post-translational modifications, such as acetylation, methylation, phosphorylation, sumoylation, and ubiquitylation [107]. These modifications, particularly at the amino-terminal histone tails, play a critical role in regulating gene expression by altering chromatin structure, shifting between open (transcriptionally active) and closed (transcriptionally repressed) conformations [84]. As histone modifications are mediated by specific enzymes, they are amenable to therapeutic modulation through the inhibition or activation of these enzymes. Among the histone modifications, acetylation and methylation have been most extensively studied, with numerous inhibitors targeting histone acetylases, deacetylases, methyltransferases, and demethylases showing promise in preclinical models of atherosclerotic CVD [108]. For instance, isoform-specific and cell-specific inhibition of histone deacetylases (HDACs) has demonstrated beneficial effects. Inhibitors of HDAC3 and HDAC9 significantly reduced atherosclerosis in mouse models [75,109]. Targeted strategies, such as using ESM-HDAC528, a pan-HDAC inhibitor designed to accumulate in monocytes and macrophages [110], shifted lesions toward a less severe phenotype [111], though it failed to reduce plaque size [110].

In individuals with atherosclerotic CVD and type 2 diabetes, genome-wide alterations in histone acetylation patterns have been observed in peripheral blood mononuclear cells [112]. For example, changes in histone 3 acetylation at lysine 9 (H3K9ac) at specific loci have implicated histone acetylation in the pathogenesis of atherosclerotic CVD in diabetes [112]. However, studies specifically exploring histone-modifying inhibitors in diabetes-associated atherosclerosis remain limited. The class I HDAC inhibitor valproate has shown potential by attenuating atherosclerosis in diabetic Apoe+ mice [113]. Furthermore, macrophages from diabetic atherosclerotic plaques exhibit elevated HDAC3 levels, which correlate with plasma lipid levels [114]. Genetic studies using the lysozyme 2 promoter-driven Cre-lox system to delete Hdac3 in myeloid cells revealed a significant reduction in atherosclerosis in Apoe+ mice on a high-fat diet [114]. However, this model is not specific to diabetes, and the system targets a broader range of myeloid cells rather than macrophages exclusively. Despite promising findings, significant gaps remain, particularly in understanding the cell-specific roles of histone modifiers, such as HDAC3, in diabetes-associated atherosclerosis.

### 2.8. Trained Immunity

Trained immunity refers to the ability of the innate immune system to develop a memory-like response to previous exposures, either to pathogens or sterile triggers, through epigenetic reprogramming [115]. This unique form of immunity can be triggered by diverse stimuli, such as oxidized LDL (oxLDL) and hyperglycemia [116,117,118]. Unlike adaptive immunity, trained immunity is non-specific and results in a sustained, heightened innate immune response to subsequent stimuli, whether similar or unrelated. This phenomenon is driven by both metabolic and epigenetic reprogramming and has been implicated as a potential mechanism underlying the chronic inflammation observed in diabetes-accelerated atherosclerosis [115,118].

Epigenetic modifications are central to trained immunity, with histone alterations such as H3K4me1, H3K4me3, H3K9me2, and H3K27ac playing critical roles in sustaining the activated immune phenotype [119,120,121]. In diabetes, hyperglycemia has been shown to induce specific epigenetic changes in monocytes, including reduced H3K4me2 levels at the IL8 gene and increased H3K9me2 levels at the IL1A gene, both associated with persistent inflammation [122]. Additionally, elevated histone acetylation levels at promoters of inflammatory genes, such as those encoding TNF-α and COX-2, have been observed in monocytes from individuals with type 1 or type 2 diabetes [123]. These epigenetic changes contribute to the pro-inflammatory and pro-atherogenic state seen in hyperglycemia.

Hyperglycemia has also been shown to induce trained immunity in macrophages [117]. Bone marrow-derived macrophages from diabetic mice retained a pro-inflammatory gene expression profile even after being cultured in normal glucose conditions, indicating a long-lasting effect of hyperglycemia [117]. Transplantation studies in non-diabetic atheroprone mice (Ldlr+) revealed that bone marrow from diabetic donors significantly increased atherosclerosis burden, further supporting the role of trained immunity in diabetes-associated atherosclerosis [117].

The transcription factor RUNX1 has emerged as a key regulator in hyperglycemia-induced trained immunity [117]. RUNX1 target genes were enriched in human atherosclerotic plaques and peripheral blood leukocytes, and pharmacological inhibition of RUNX1 attenuated trained immunity responses in macrophages, highlighting its potential as a therapeutic target [117]. Further research into additional pharmacological targets for modulating trained immunity in this context is warranted.

## 3. Established Therapeutic Approaches

**Figure 2:**
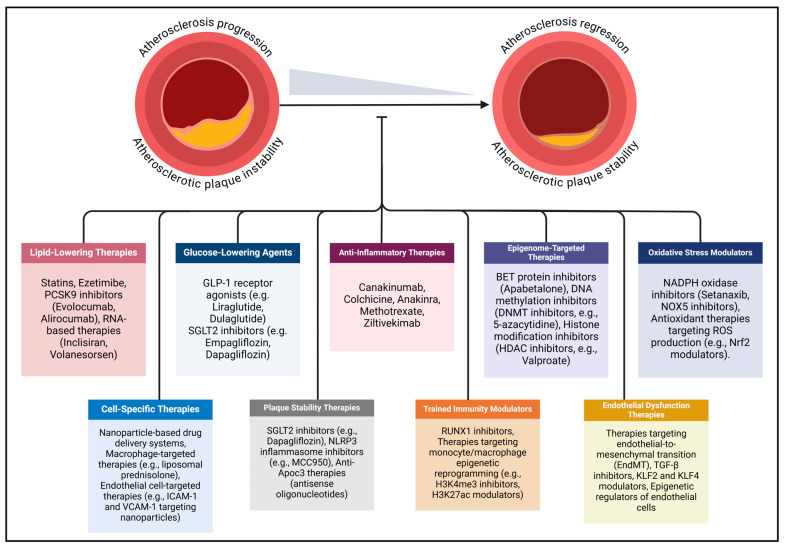
Emerging mechanism-based therapeutic strategies for diabetes-associated atherosclerosis are gaining attention due to limitations in current treatments for atherosclerotic cardiovascular disease. Existing therapies primarily address conventional risk factors, such as hyperglycemia, hyperlipidemia, and hypertension, but fail to adequately target the unique pathophysiological mechanisms underlying atherosclerosis in diabetes mellitus. Novel approaches focus on anti-inflammatory therapies, epigenome-targeted therapies, oxidative stress modulators, cell-specific therapies, endothelial dysfunction therapies, plaque stability, trained immunity modulators, and targeted molecular therapies. The dash at the end of the line indicates inhibition. Abbreviations: ApoC3 (apolipoprotein C-III), BET (bromodomain and extra-terminal protein), DNMT (DNA methyltransferase), EndMT (endothelial-to-mesenchymal transition), GLP-1 (glucagon-like peptide-1), ICAM-1 (intercellular adhesion molecule-1), KLF (Kruppel-like factor), MCC950 (specific NLRP3 inflammasome inhibitor), Nrf2 (nuclear factor erythroid 2-related factor 2), NLRP3 (NOD-, LRR-, and pyrin domain-containing protein 3), NOX (NADPH oxidase), PCSK9 (proprotein convertase subtilisin/kexin type 9), ROS (reactive oxygen species), RUNX1 (runt-related transcription factor 1), SGLT2 (sodium–glucose cotransporter 2), TGF-β (transforming growth factor-beta), VCAM-1 (vascular cell adhesion molecule-1).

In diabetes, the accelerated progression of atherosclerosis extends beyond conventional cardiovascular risk factors such as dyslipidemia and hypertension, with hyperglycemia, oxidative stress, and inflammation playing central roles. To address these risks, contemporary clinical strategies focus on managing atherosclerotic CVD through targeted control of hypercholesterolemia, hyperglycemia, and hypertension (Figure 2). In individuals with diabetes, this approach is further refined with a multifaceted therapeutic regimen that includes lipid-lowering medications, glucose-lowering therapies, antihypertensive treatments, and emerging anti-inflammatory interventions.

### 3.1. Lipid-Lowering Medications

Cholesterol-lowering therapies, including statins, ezetimibe, and PCSK9 inhibitors, are widely employed to reduce plasma LDL cholesterol levels. Their combined use has demonstrated enhanced efficacy, as evidenced by the IMPROVE-IT trial [124], which reported further reductions in LDL cholesterol levels and improved cardiovascular outcomes with the addition of ezetimibe to statin therapy compared to statins alone. Beyond their lipid-lowering properties, statins exhibit pleiotropic effects, including anti-inflammatory actions, as reflected by reductions in plasma C-reactive protein (CRP) levels [125]. Notably, an analysis of three randomized statin trials highlighted that inflammation and high-sensitivity CRP (hsCRP) levels are stronger predictors of future cardiovascular events than plasma LDL cholesterol levels in individuals undergoing statin therapy [126].

The FOURIER and ODYSSEY OUTCOMES trials demonstrated that treatment with the anti-PCSK9 monoclonal antibodies evolocumab and alirocumab significantly improved cardiovascular outcomes compared to placebo [127,128]. A meta-analysis of randomized controlled trials further confirmed that both agents are well tolerated [129]. Subgroup analyses from the ODYSSEY OUTCOMES, FOURIER, and PROFICIO phase III trials revealed substantial reductions in plasma LDL cholesterol levels among patients with diabetes receiving PCSK9 inhibitors compared to placebo [130]. Additional evidence from trials such as ODYSSEY DM-INSULIN and ODYSSEY DM-DYSLIPIDEMIA highlighted the efficacy of PCSK9 inhibitors in individuals with hypercholesterolemia and diabetes [131,132]. When added to maximally tolerated statin therapy, PCSK9 inhibitors significantly reduced plasma LDL cholesterol levels and prevented cardiovascular events without adversely affecting glycemic control, offering a robust therapeutic option beyond statin therapy alone [131,132,133,134].

Emerging therapies for dyslipidemia focus on RNA-based gene-silencing technologies, including small interfering RNAs (siRNAs) and antisense oligonucleotides (ASOs). siRNAs are double-stranded RNA molecules that induce the degradation or translational inhibition of target mRNAs via the RNA-induced silencing complex, while ASOs are single-stranded RNA sequences complementary to target mRNAs, preventing their translation into proteins. These nucleic acid therapeutics primarily target genes involved in lipoprotein metabolism, such as PCSK9, apolipoprotein (a), apolipoprotein B, angiopoietin-related proteins 3 and 4, and apolipoprotein C-III (APOC3).

To enhance liver-specific delivery and minimize systemic toxicity, most RNA-based therapeutics are conjugated with N-acetylgalactosamine (GalNAc) [135]. One such therapy, inclisiran, a GalNAc-conjugated siRNA targeting PCSK9, has been approved for treating adults with heterozygous familial hypercholesterolemia or clinical atherosclerotic CVD [135]. Inclisiran’s twice-yearly dosing schedule offers a significant advantage over the more frequent regimens of monoclonal PCSK9 inhibitors, potentially improving adherence. In the ORION-10 and ORION-11 trials, inclisiran demonstrated substantial reductions in plasma LDL cholesterol levels by 52.3% and 49.9%, respectively, compared to placebo [135]. Post hoc analyses of the ORION-9, ORION-10, and ORION-11 trials further showed that inclisiran significantly decreased PCSK9 levels and other atherogenic lipids in patients with diabetes or obesity [135,136]. Another promising RNA-based therapy is volanesorsen, a GalNAc-conjugated ASO targeting APOC3 mRNA [137]. Approved by the EMA for patients with familial chylomicronemia at high risk of pancreatitis, volanesorsen has also demonstrated efficacy in patients with type 2 diabetes by significantly lowering fasting plasma APOC3 and triglyceride levels while improving insulin sensitivity and glycemic control compared to placebo [137].

### 3.2. Glucose-Lowering Agents

GLP-1 receptor agonists and SGLT2 inhibitors, newer therapeutic agents approved for type 2 diabetes, have demonstrated not only significant glucose-lowering effects but also notable extra-glycemic effects, including reductions in cardiovascular events [138,139,140,141,142,143,144,145,146,147,148,149]. Remarkably, these cardioprotective benefits have also been observed in individuals with heart failure, including those without diabetes or kidney disease [138,139,140,141,142,143,144,145,146,147,148,149]. GLP-1, an incretin hormone secreted by intestinal L cells in response to food intake, enhances glucose-dependent insulin secretion via GLP-1 receptor activation on pancreatic islets [150]. Beyond its pancreatic actions, emerging evidence highlights the extra-pancreatic roles of GLP-1 [150]. Experimental studies on GLP-1 receptor agonists [151,152] and clinical trials, including LEADER and REWIND [152,153], which investigated liraglutide and dulaglutide, respectively, have revealed both renoprotective and cardioprotective effects of GLP-1 receptor activation. However, further investigation is needed to determine the extent to which these benefits are independent of glucose regulation.

The cardioprotective benefits of SGLT2 inhibitors have been observed even in individuals who are lean, normotensive, and non-diabetic, suggesting that their mechanisms of action extend beyond glucose regulation, weight reduction, and blood pressure control [154,155,156]. In preclinical studies, SGLT2 inhibitors improved cardiac function in a mouse model of heart failure, including in Sglt2-deficient mice, indicating off-target effects and benefits independent of metabolic alterations [157]. Despite substantial evidence supporting the multisystem benefits of SGLT2 inhibitors, the precise mechanisms underlying these effects, beyond their glucose-lowering properties, remain incompletely understood. Notably, clinical evidence suggests that GLP-1 receptor agonists exhibit stronger efficacy than SGLT2 inhibitors in reducing atherosclerosis-related cardiovascular events, such as myocardial infarction and stroke [157]. Experimental studies indicate that SGLT2 inhibitors may exert anti-atherosclerotic effects by improving endothelial dysfunction and reducing vascular injury [158]. However, robust clinical data confirming these findings are still lacking. Current clinical guidelines recommend SGLT2 inhibitors for patients with heart failure or kidney disease, while GLP-1 receptor agonists are advised for individuals with type 2 diabetes at high risk of atherosclerotic CVD or stroke, as well as for those with kidney disease [159].

### 3.3. Anti-Inflammatory Therapies

Large-scale, randomized clinical trials have provided compelling evidence for the role of inflammation in the pathogenesis of atherosclerosis and the potential benefits of anti-inflammatory therapies in atherosclerotic CVD. The phase III CANTOS trial [160] demonstrated that treatment with canakinumab, a monoclonal antibody targeting IL-1β, significantly improved cardiovascular outcomes in patients with a history of myocardial infarction. However, the increased risk of fatal infections associated with canakinumab has precluded its approval for cardiovascular indications. Colchicine, a broad-spectrum anti-inflammatory agent that inhibits the NLRP3 inflammasome, has emerged as a promising therapy for atherosclerosis prevention. The COLCOT trial [161] demonstrated the efficacy of colchicine in reducing cardiovascular events in post-myocardial infarction patients, while the LoDoCo2 trial [162] confirmed its benefits in patients with chronic stable coronary artery disease. Based on these findings, colchicine was approved by the FDA in 2023 for the treatment of atherosclerotic CVD [163]. Additionally, a meta-analysis encompassing the landmark trials revealed that anti-inflammatory therapies in patients with type 2 diabetes were associated with a significant reduction in the risk of major adverse cardiovascular events compared to placebo, underscoring the therapeutic potential of targeting inflammation in this high-risk population [164].

Anakinra, a human recombinant IL-1 receptor antagonist, has been investigated in patients with ST-segment elevation myocardial infarction to assess its impact on systemic inflammation, using hsCRP as a primary marker [165]. While results demonstrated a short-term reduction in inflammation compared to placebo, anakinra has not received approval for treating atherosclerotic CVD [165].

Methotrexate, a purine metabolism inhibitor commonly used for immunosuppression in conditions like rheumatoid arthritis, was evaluated in the CIRT trial [166]. This trial investigated the effects of low-dose methotrexate on cardiovascular outcomes in patients with multivessel coronary disease, or those with a history of myocardial infarction and type 2 diabetes or metabolic syndrome. However, the low-dose methotrexate failed to reduce plasma levels of IL-1β, IL-6, or hsCRP and did not improve outcomes in patients with stable atherosclerosis, limiting its potential utility in this context [166].

Ziltivekimab, a monoclonal antibody targeting IL-6, showed superior anti-inflammatory effects in the RESCUE trial for CKD patients with elevated hsCRP compared to IL-1β inhibition in the CANTOS trial [167]. It is currently under evaluation in the ZEUS trial for atherosclerotic CVD in CKD patients [168] and the HERMES trial for its impact on heart failure-related outcomes [169].

## 4. Therapeutic Strategies Under Investigation

Despite advancements in mitigating conventional CVD risk factors through existing therapies, a significant residual risk persists, particularly among individuals with diabetes, where many patients continue to experience recurrent cardiovascular events [170]. Moreover, certain therapies are associated with adverse effects in specific patient populations [171]. This highlights an urgent need for novel strategies to address these unmet clinical challenges effectively.

### 4.1. Targeted Cellular Therapies

Systemic drug administration is often hindered by several limitations, including low bioavailability, reduced stability, rapid clearance, and off-target effects [172,173]. Targeted drug delivery to specific cells at the site of injury offers a promising alternative to overcome these challenges. However, implementing cell-specific therapies in atherosclerosis remains complex [172,173]. Effective strategies must account for the dynamic roles of various cell populations throughout the stages of atherogenesis and target antigens uniquely expressed on affected cells in a time-sensitive manner [172,173]. The choice of drug carriers plays a critical role in the success of targeted therapies. Nanoparticles are the most widely utilized carriers due to their ability to enhance drug stability, bioavailability, and precision delivery to the site of injury while minimizing off-target effects [172,173]. Furthermore, nanoparticles allow for controlled, sustained drug release, which is particularly beneficial in conditions like diabetes. For instance, glucose-lowering drugs, such as GLP-1 receptor agonists, can be encapsulated in nanoparticles to mimic the natural secretion of GLP-1 in response to food intake [172,173]. Current nanocarriers include polymer-based nanoparticles, HDL-like and LDL-like nanoparticles, and liposomes, each offering unique advantages for improving drug delivery efficiency and specificity [172,173]. These innovations represent significant progress toward overcoming the limitations of systemic therapies and advancing precision medicine in atherosclerosis and other chronic diseases.

Nanoparticles have been investigated in clinical trials for individuals with diabetes, although their application in the context of atherosclerotic CVD remains unexplored [174,175,176]. Considering that atherosclerosis in diabetes involves similar cellular mechanisms as in non-diabetic individuals, the development of nanotherapeutics for atherosclerosis may benefit both populations. The roles of various vascular cell populations vary across the different stages of atherosclerotic plaque progression [174,175,176]. Advances in cell-specific technologies have enhanced the understanding of cellular heterogeneity among vascular and immune cells. However, a unified classification system for subpopulations, such as endothelial cells and macrophages, has yet to be established. Injured vascular cells express unique surface proteins that can be exploited for targeted drug delivery systems [174,175,176]. For instance, chronically activated endothelial cells express markers like intercellular adhesion molecule-1 (ICAM-1), vascular cell adhesion molecule-1 (VCAM-1), platelet endothelial cell adhesion molecule (PECAM-1), E-selectin, and P-selectin, all of which represent potential targets for nanoparticle-based therapeutic interventions [177,178,179].

Macrophages are pivotal immune regulators in atherosclerotic plaques [180], with therapeutic strategies targeting pathways such as inflammation and impaired cholesterol efflux [181]. Preclinical studies have identified macrophage-specific targets, including calcium–calmodulin-dependent protein kinase IIγ, epsins, and the CD47–signal regulatory protein-α axis [182,183]. However, the recent discovery of diverse macrophage subpopulations, revealed through technologies like single-cell RNA sequencing, underscores the complexity of targeting specific macrophage populations. Macrophage-directed nanotherapies have demonstrated promise in preclinical models by improving therapeutic precision and reducing off-target effects [180]. For instance, liposomal nanoparticles encapsulating prednisolone successfully targeted macrophages in patients undergoing endarterectomy for symptomatic atherosclerosis [184]. Additionally, research into VSMC has highlighted potential targets such as the transient receptor potential cation channel TRPV1, which reduces foam cell formation via autophagy induction [185]. In Apoe+ mice, copper sulfide nanoparticles carrying monoclonal antibodies to TRPV1, activated by near-infrared light, decreased lipid accumulation in VSMCs and reduced atherosclerosis [186]. Other VSMC-specific targets, including transcription factor 21, hyaluronan synthase 3, KLF4, growth differentiation factor 10, and NOTCH, offer potential therapeutic avenues for atherosclerosis, including in diabetes [187].

### 4.2. NADPH Oxidase Inhibitors

Setanaxib, a dual NOX1-NOX4 inhibitor, is the first NADPH oxidase inhibitor to reach clinical trials. Preclinical studies demonstrated its dual renoprotective and atheroprotective effects in Apoe+ mice with STZ-induced diabetes, with NOX1 inhibition reducing atherosclerosis and NOX4 deletion unexpectedly increasing plaque area [19,20]. The balanced inhibition of NOX1 and NOX4 by setanaxib appears to mediate its protective effects [188]. Although setanaxib is under investigation for conditions such as idiopathic pulmonary fibrosis [189], primary biliary cholangitis [190], and squamous cell carcinoma [190], its trial in type 2 diabetes-associated kidney disease failed to achieve the primary endpoint of albuminuria reduction. While its long-term cardiovascular benefits remain unproven, setanaxib’s consistent anti-inflammatory effects suggest potential cardioprotective properties.

The upregulation of NOX5 is implicated in several pathological conditions, including hypertension, diabetes, inflammation, and fibrosis, making it a promising target for improving cardiovascular and renal outcomes in diabetes [191]. NOX5 is expressed in all vascular wall cell types and plays a role throughout atherosclerosis progression [30]. Epigenetic mechanisms regulate NOX5 expression, as shown in studies where histone acetyltransferases, particularly p300 and HAT1, promoted NOX5 overexpression under inflammatory conditions [192]. Inhibition of histone deacetylases (HDACs) suppressed NOX enzyme transcription, reduced ROS production, and improved outcomes in models of pulmonary arterial hypertension and diabetes [193,194]. In vitro, high glucose levels enriched H3K27ac at the promoters of NOX1, NOX4, and NOX5 in human VSMCs, further linking HDAC activity to NOX-mediated vascular dysfunction [194]. These findings suggest that targeting HDACs could offer a novel therapeutic approach for diabetes-associated atherosclerosis.

### 4.3. Epigenome-Targeted Therapies

Inhibitors of enzymes involved in epigenetic regulation represent a promising frontier in pharmacology, with advances in chromatin biology enabling the development of novel therapies targeting DNA methylation and histone modifications [195]. These drugs modulate epigenetic marks to influence gene expression linked to diseases such as atherosclerosis [196].

Apabetalone (RVX-208), the first bromodomain and extra-terminal (BET) protein inhibitor to receive FDA breakthrough therapy designation, has shown potential for reducing major adverse cardiovascular events (MACEs) in patients with type 2 diabetes [197]. BET proteins (BRD2, BRD3, BRD4, and BRDT) act as epigenetic readers, facilitating transcription by binding acetylated histones [198]. Preclinical studies suggest that BET inhibition reduces atherosclerosis, stimulates reverse cholesterol transport by increasing ApoA-I expression and HDL levels, and attenuates hyperglycemia-induced inflammation [199,200]. In Apoe+ mice and human endothelial cells, apabetalone suppressed inflammatory markers like IL-1β, IL-6, and TNF-α [201]. Clinical studies also indicate reduced systemic inflammation and fewer cardiovascular events with apabetalone treatment compared to placebo [202].

The phase III BETonMACE trial demonstrated benefits for secondary endpoints, including reduced hospitalization for heart failure in patients with type 2 diabetes and recent acute coronary syndrome [197]. Building on these findings, the upcoming BETonMACE2 trial will evaluate the combined effects of apabetalone and an SGLT2 inhibitor to further investigate its cardiovascular benefits.

### 4.4. Resolution-Based Therapies

Emerging evidence suggests that impaired resolution of inflammation plays a crucial role in the progression of atherosclerosis, particularly in the context of diabetes [203]. Unlike traditional anti-inflammatory therapies that primarily suppress immune activation, resolution-based therapies actively promote the resolution of inflammation and restore vascular homeostasis [203]. These approaches leverage specialized pro-resolving mediators (SPMs), such as resolvins, maresins, and lipoxins, to counteract persistent inflammation, enhance efferocytosis, and stabilize atherosclerotic plaques [204,205,206,207].

Resolvins, particularly from the RvD and RvE series, have been shown to mitigate vascular inflammation by reducing pro-inflammatory cytokine expression and promoting macrophage polarization toward an anti-inflammatory phenotype [204,205]. Maresins, including MaR1 and MaR2, play a crucial role in inhibiting endothelial dysfunction and preventing foam cell formation, thereby limiting the progression of atherosclerotic plaques [207]. Similarly, lipoxins, such as LXA4 and LXB4, contribute to vascular protection by suppressing leukocyte recruitment, limiting neutrophil infiltration, and reducing oxidative stress, ultimately improving endothelial function [206]. Collectively, these specialized mediators facilitate the resolution phase of inflammation and promote tissue homeostasis, distinguishing them from conventional anti-inflammatory agents that primarily inhibit inflammatory responses without actively resolving them.

Beyond the direct administration of SPMs, biomimetic nanoparticles have emerged as a promising strategy for targeted drug delivery, enhancing the bioavailability and therapeutic efficacy of resolution-promoting mediators [208,209]. These nanoparticles can be engineered to mimic endogenous biological structures, allowing for improved targeting of inflamed vascular sites while minimizing systemic side effects [208,209]. Lipid-based nanoparticles have been developed to encapsulate and release SPMs in a controlled manner, sustaining their biological effects over time [210,211]. Polymeric nanoparticles provide an additional strategy for precise drug release, improving the therapeutic window of anti-inflammatory agents. Furthermore, cell membrane-coated nanoparticles, which leverage platelet- or macrophage-derived membranes, have been shown to enhance selective accumulation at sites of vascular inflammation [211,212].

Preclinical studies have demonstrated that nanoparticle-based delivery of resolvins, maresins, and lipoxins enhances their stability and efficacy, ultimately reducing vascular inflammation and promoting plaque stability [213]. Given the chronic inflammatory nature of diabetes-driven atherosclerosis, resolution-based therapies represent a promising paradigm shift by addressing inflammation while avoiding the immunosuppressive effects associated with traditional anti-inflammatory agents [214,215]. Future research should focus on optimizing the stability, bioavailability, and targeted delivery of these mediators. Additionally, clinical trials assessing the long-term safety and efficacy of SPMs and nanoparticle-based therapies will be essential in translating these findings into therapeutic interventions for patients with diabetes and cardiovascular disease.

## 5. Conclusions

Atherosclerosis in diabetes represents a complex interplay of metabolic, inflammatory, and epigenetic mechanisms that accelerate disease progression and enhance plaque vulnerability. Despite advancements in conventional therapeutic strategies targeting dyslipidemia, hyperglycemia, and hypertension, residual cardiovascular risk remains substantial, necessitating innovative approaches. Emerging therapies, such as targeted nanomedicine, epigenetic modulators, and mechanism-based interventions, provide promising avenues for mitigating diabetes-associated atherosclerosis. Novel glucose-lowering agents, including SGLT2 inhibitors and GLP-1 receptor agonists, extend benefits beyond glycemic control, addressing cardiovascular and renal complications. Additionally, cell-specific therapies and advances in RNA-based technologies herald a paradigm shift toward precision medicine.

The integration of these cutting-edge therapies with traditional strategies has the potential to address the unmet clinical need for reducing recurrent cardiovascular events in diabetes. However, translating preclinical findings into effective clinical applications will require further robust investigations. A multidisciplinary approach leveraging systems biology, multiomics, and advanced drug delivery platforms is essential to unravel the complexity of diabetes-driven atherosclerosis and develop tailored therapeutic solutions for this high-risk population.

## Data Availability

No new data were created or analyzed in this study.
